# Supplementary material for: Are carbon emissions trading and green financial instruments synergistic? -Comprehensive quantitative research based on content analysis
Source: PLoS One. 2024 Mar 7;19(3):e0298601. doi: 10.1371/journal.pone.0298601 (PMC10919601; doi:10.1371/journal.pone.0298601)
Supplement: S1 Data — (ZIP) [file pone.0298601.s001.zip › Data/地级市Z_F工作报告.zip!/╜¡╬≈/─╧▓2╩╨/└┤╫╘╣1⁄2╓┌║┼í╛┬φ┐╦╩2╛▌═°í┐_2003.docx]

各位代表：

　　现在，我代表市人民政府，向大会作政府工作报告，请予审议，并请各位政协委员和其他列席人员提出意见。

　　一、2002年工作简要回顾

　　过去的一年，在省委、省政府和市委的正确领导下，全市人民高举邓小平理论伟大旗帜，努力实践“三个代表”重要思想，紧紧围绕“富民强市”目标，全力做好“两篇文章”，强力推进“三个率先”，创造了“一派生机、一片商机、一方沃土”的发展环境，营造了“上下同志谋发展，左右同力干事业，异口同声创辉煌”的发展氛围，呈现了“人心思上、事业兴旺、经济加速、城乡变样”的发展势头。通过努力，南昌“做优了环境，做旺了人气，做活了商机，做强了实力，做大了名声”，全面完成了市十二届人大二次会议确定的工作任务。

　　国内生产总值552亿元，增长13.8％；

　　全社会固定资产投资137亿元，增长41％；

　　财政总收入60.8亿元，增长16％；

　　地方财政收入27.4亿元，增长22.1％；

　　实际利用外资3.6亿美元，增长2倍；

　　实际利用内资65亿元，增长70.2％；

　　社会消费品零售总额179.3亿元，增长11.5％；

　　城市居民年人均可支配收入7000元，增长12.8％；

　　农民年人均纯收入2667元，增长6％；

　　人口出生率1462‰。

　　在建设现代区域经济中心城市上，坚持以大开放为主战略，以工业化为核心，狠抓工业、农业、三产、招商引资、个私民营经济五个着力点，进一步增强了南昌的聚集力、辐射力、带动力。

　　第一，工业经济呈现出“发展速度逐月上升，骨干企业增势强劲，企业改制步伐加快，企业上市取得突破”的良好态势。立足“做大做强骨干企业，做专做精特色企业，放开搞活中小企业”，狠抓“三个一批”：即聚酯切片、数字彩电等一批重点骨干项目，银志服装、格林柯尔等一批产业梯度转移项目，泰丰轮胎、汇仁药业、江纺、江铃等一批技术改造项目；突出“四个重点”：即发展高新产业，争创名牌产品，培植企业集团，建设工业园区。全市现有高新企业近200家，全国驰名商标2个，省著名商标36个，省名牌产品40个，上市公司12家，县级以上工业园区13个。特别是开发园区，抓现的、上快的、盯着有后劲的，区区你追我赶，园园热火朝天，成为“南昌境内盏盏明灯，花园城市朵朵奇葩”。高新技术产业区加速崛起，累计进区企业620家，其中高新技术企业163家。经济技术开发区平均一月引进10个项目，一月开发1平方公里，誉为“南昌的浦东，深圳的速度”。红谷滩新区聚行政、居住、商务于一身，融环境、文化、服务于一体，进区项目47个，投资总额167亿元。民营科技园越做越大，杭州娃哈哈、银志服装城等一批大项目落户，已成为“全国十佳民营科技园”之一。昌东工业园发展迅速，规划面积达21.5平方公里。小兰工业园势头强劲，引进了上海绿地、厦门建发、清华泰豪等一批知名企业。长?工业区红红火火，引进项目74个，实际进资78亿元。昌南工业园脱颖而出，进园企业28家，引进资金787亿元。通过努力，全市工业总产值、工业增加值分别增长19％和176％；41户重点企业份额增大；104户国有中小企业完成改制；3户企业成功上市，23户企业准备上市。

　　第二，农村工作呈现出“农业增效，农民增收，负担减轻，社会稳定”的良好局面。按照“土地向大户转移，人口向城镇转移，企业向园区转移”的工作思路，突出“十抓”：一是抓调整，水稻面积调减1375万亩，经济作物比重超过45％；二是抓订单，全市农业订单金额5亿元，覆盖农户20％。成功举办了首届“中国·南昌鄱阳湖螃蟹节”，签订农业项目40个，合同资金638亿元；三是抓龙头，市级产业化龙头企业40家，年产值超千万元的新增6家，超亿元的新增3家。南昌（深圳）农产品中心批发市场被评为国家级龙头企业；四是抓园区，县乡工业园区初具规模，开发面积604平方公里，投资规模73亿元。建起了蒋巷现代农业示范园、扬子洲现代生态农业科技园、安义高新农业科技园、进贤中华绒鳌蟹繁育示范园；五是抓流通，各类专业合作经济组织62个，年营销额10万元以上的农村商品经纪人232人；六是抓市场，水产品综合批发市场、豪威花鸟大市场已开工建设；七是抓能人，成立了“农村创业者俱乐部”，派遣了10名农村致富带头人赴日本研修学习；八是抓基础，加强农田水利基本建设，实施退田还湖、退耕还林、移民建镇工程。强化农村公路升级改造，完成干道路面硬化230公里；九是抓改革，税费改革进展顺利，广大农民得到实惠，中央、省、市、县区四级财政转移支付102亿元，全市农村人均减负3953元。十是抓致富，村支书“双带”致富工程初见成效，“一村一品”工程建设进展顺利，发展各类专业村112个；对50个重点贫困村开展综合帮扶，实施扶贫开发项目116个。同时，成功扩销高价库存粮7.2亿斤，节约财政风险金700万元。

　　第三，第三产业呈现出“商贸流通焕发生机，房地产业持续升温，旅游产业发展强劲”的良好格局。商贸业按照“城内大商场，城郊大市场，城中专业街，城外大物流”的布局，品牌专业街形成特色：抚河路（沿江路）综合商业街，争相开张；胜利路百年老街，重放光彩；中山路传统商业街，发扬光大；孺子路餐饮街，顾客盈门。大商场销售火爆：百货大楼、洪城大厦等大型零售商场均以两位数增长；太平洋百货、华联超市、深圳天虹、沃尔玛、麦当劳等一批知名企业，纷纷抢滩南昌；宁波金润、厦门建发等一批大型“物流中心”，纷纷进驻南昌。大市场交易活跃：年交易额超亿元商品市场发展到25个，交易额保持两位数增长。洪城大市场年交易额突破100亿元。房地产业“投资增长，供需两旺，品质提升，楼盘增值”，形成了控制总量、有序开发、激活市场、促进升值的“南昌模式”，全市商品房销售额增长41％，二手房销售额增长115％。旅游业采取“政府主导、整体组合、多条渠道、多方投入”举措，组建“南昌旅游集团”，唱响“五彩缤纷看南昌”主题，成功举办首届绳金塔文化经贸节、食在南昌“美食周”等会展活动，“新豫章十景”评选活动，全年游客增长15％，旅游总收入增长286％。

　　第四，招商引资呈现出“外资规模越来越大，项目水平越来越高，进资速度越来越快，发展后劲越来越强”的鲜明特点。营造诚信环境，打造“四最”品牌，放开投资领域，创新招商手段，实行全方位开放，大力度招商。在境内，实行北上、南下、东进，举办了“北京恳谈会”、“深圳招商会”、“温州推介会”、“厦门洽谈会”、“全国知名民营企业家南昌项目洽谈会”等系列招商活动。在境外，突出港台，开拓欧美，开展了香港大规模招商，国外小分队招商。特别是去年九月成功举办的“中国南昌首届金秋经贸活动月”，在我市历史上，时间最长、规模最大、内容最丰富、成果最显著，签约项目多达160个，其中超亿元项目13个。

　　第五，个私民营经济呈现出“多路挺进、多业并举、多点延伸”的发展之势。坚持“六放方针”，做到“八个同等”，民间投资空前高涨，企业个数与日俱增，规模壮大，总量攀升，已近“三分天下有其一”。全市净增个私民营企业1100家，净增注册资金38亿元；个私民营企业增加值增长279％，占全市GDP比重达到283％，上升了32个百分点。

　　在创建现代文明花园英雄城市上，坚持高起点规划，高质量建设，高水平管理，高效益经营，进一步做强、做大、做优、做美了南昌。

　　第一，坚持名人指点、高手过招、领导审查、市民参与，高水平规划新南昌。按照到2010年的城市发展目标，展开“一江两岸，南北双城，沿着赣江，两头延伸，内外两环，外带五大组团”规划。南昌城市规划展示馆已免费向市民开放，《南昌市城市总体规划（2002～2020）》正在加紧编制。城市控制性规划由6％提高到40％，红谷滩新区控制性规划达到100％。

　　第二，坚持改造老城区、建设新城区、储备朝阳区，高标准建设新南昌。昌南，拆建旧城换新颜：城建、防洪统一规划，道路、圩堤融为一体，环境设施综合配套，东西南北六十公里（防洪圈、外环路）。坚持“居民住宅原则不建，工业企业退二进三，发展商贸，繁荣三产，优化环境，市民休闲”，突出道路维修、管线规范、功能完善、街道改造，完成了苏圃路、环湖路、广场南路、系马桩街、百花洲横街、何坊西路改造工程，进行了地王广场、胜利广场、章江路等片区的危旧低洼房改造。昌北，新建新城展新貌：背靠西山山脉，面对浩瀚赣江，挖掘文化底蕴，做好山水文章，建筑各具特色，一派都市风光。重点把握“三线”（梅岭、乌石河、赣江），启动“三片”（按生活居住区规划凤凰洲，按综合功能区打造红角洲，按中央商务区建好红谷滩），美化“三湖”（碟子湖、前湖、黄家湖），注重“三点”（高校园区、工业园区、港口码头），同时搞好瀛上墓地搬迁。红谷滩新区路网工程进展顺利，世纪花园、丰和花园、住宅小区、报业大厦、万达星城、红谷春天、南昌五中、世贸中心全面启动，一批标志性建筑先后动工。

　　在城市基础设施完善上，实施了胡惠元堤、洪都大道、高新大道、昌东大道、朝阳洲主干道等城市道路骨架工程；完成了青山湖清淤截污及周边美化，抚河整治换水，富大有堤、赣东大堤除险加固工程；正在实施玉带河综合治理工程；启动了青山湖污水处理厂、青云水厂三期工程、人防指挥救援中心、道路照明自动监控系统工程建设。加大城市供水、供气管网改造力度，实现新建、改建项目管线统一下地。

　　第三，坚持经营城市新理念，高效益经营南昌。强化土地储备中心功能，组建三大公司（土地经营公司、城建投资公司、市政投资公司），着力盘活“三块”资源（城市土地资源、城市基础设施、城市无形资产），全市土地出让金额突破47亿元，政府净收益超过8亿元。同时，做好了土地定级估价、城市基准地价工作，建立了全市地价动态监测系统。经营性土地招标拍卖、挂牌交易工作成效显著。

　　第四，坚持突出工作重点，高品位塑造南昌。推进“六大工程”（环境整治、城市绿化、美化亮化、为民造福、社会文明、长效管理），实现“四增”目标（增绿、增亮、增色、增净）。一是依托江、河、湖、园林广场及景点配套建设增绿。全年植树370万株，完成绿地面积151.5公顷，拆墙透绿56万平方米，腾地建绿21万平方米，新铺草皮66.5万平方米。二是围绕江河、湖泊、高楼、亭阁、桥梁、绿地增亮。在标志建筑物上打造灯光点，在景观大道上构筑灯光带，在重点地段营造灯光群，新增各类灯饰3万盏。三是实行“一路一色、一路一景、色彩协调、轮廓清晰”增色。“四湖”周边、滕王阁周边、绳金塔周边，以及叠山路、沿江路、湖滨南路、何坊西路、迎宾大道等12条道路两旁建筑物，全部“穿衣戴帽”。四是立足长效管理增净。健全“两级政府、三级管理、四级网络”管理机制，狠抓八项管理制度落实，加大“两个辐射”力度，组建了市容监督员队伍，“双创”活动成果进一步巩固。

　　坚持两个文明一起抓、两篇文章一起做，协调发展各项社会事业，努力提高南昌城市文明程度和市民素质。

　　精神文明建设更加深入。紧紧围绕“优良的秩序，优美的环境，优秀的品德，优质的服务”主题，努力塑造文明城市新形象。以“2530工程”为重点，贯彻《公民道德建设实施纲要》，推出了“百名文明市民标兵”、“十大道德标兵”。去年10月，我市跨入了“全国创建文明城市先进市”行列。东湖区公园街办上营坊社区被中央文明办、民政部确定为全国文明社区示范点。

　　民主法制建设得到加强。全面完成政府机构改革，全市各级政府工作部门由248个精简为177个，精简286％；市县乡人员编制总体精简了20％。大力推进政府行政审批制度改革，削减行政审批事项505项，占55％；全市仅保留行政审批事项408项，为全国最少的城市之一。严格依法行政，强化执法监督。着眼从源头上遏止腐败，廉政建设不断加强。认真执行人大及其常委会的决议、决定，自觉接受人大法律监督、工作监督和政协民主监督。深入开展全民普法教育，大力推进依法治市。积极开展社会治安综合治理，严厉打击各类刑事犯罪和经济犯罪活动，加大对“法轮功”等邪教组织的打击力度，做好练功人员转化工作。创建“安全边际长廊”，实施“道路畅通工程”，全市治安状况良好，群众安全感大为增强。

　　维稳工作取得实效。高度重视信访工作，倾听群众呼声，实实在在为群众解决困难和问题，信访总量稳中有降，集体上访明显下降。安全生产坚持依法监察，突出专项整治，注重隐患整改，实现了“两杜绝、一减少”目标（杜绝生产经营性特大事故、校园亡人事故，减少一般事故），被评为全国安全生产先进单位。

　　社会保障工作成效显著。把下岗职工再就业作为“头等大事”、“一号工程”来抓，把一切优惠让给下岗职工，努力使下岗职工下岗不失业、分流不回家，举办了“外向企业万人招聘洽谈会”、“南昌市2002年秋季系列招聘洽谈会”等系列活动，全年安置下岗职工32万人。三条保障线进一步巩固。全市参加养老保险的企事业单位9834家，参保职工53.05万人，18万名离退休人员养老金和进中心的下岗职工基本生活保障费均按时足额发放。对协议期满的下岗职工，实行“养到老、办协保、进失保”办法，保障基本生活。弱势群体倍受关爱，城镇低保工作取得突破性进展，基本实现应保尽保，保障标准有所提高。

　　各项社会事业协调发展。教育方面，小学、初中入学率分别达到996％和942％，高考录取率达到70％。南昌教育信息中心网基本完成，10所花园式学校初具规模。积极推进教育改革，着手整合教育资源。昌北、昌东两个高校园区基础设施建设全面启动，南大、师大、南航等新校园建设全面动工。科技方面，我市已被列为全国50个制造业信息化工程重点城市之一、22个专利试点城市之一。打造人才品牌，构筑人才高地。与江西师范大学等8所高校建立了科技合作关系，清华、北大、浙大等科技园先后落户我市，清华泰豪在我市建起了首家博士后工作站。文化方面，创作了一批反映时代精神的文化精品，加大了文化市场管理力度。完成广播电视专用网750兆升级改造，全市实现光缆联通对接。体育方面，在十一届省运会上，取得了金牌总数、奖牌总数、团体总分“三个第一”；在全国残疾人特奥会上，获金牌总数第四；在国际比赛上，取得海模C5级世界冠军，获金牌4枚、银牌3枚，实现了海模C5级“三连冠”。卫生方面，加强了对重点传染病、血吸虫病、地方病的监控，全市传染病疫情基本平稳。全面实施名医、名科、名院“三名”工程。成立慈善医院，开展医疗救助。

　　可持续发展战略深入人心。认真落实计划生育基本国策，强化城市流动人口、特殊人群计生管理，开展优生优育服务，提高出生人口素质。重视生态环境保护，加强综合治理，积极实施退耕还林、长江保护林、生态公益林等工程建设，造福社会民众。

　　同时，广泛开展国防教育，提高全民国防意识。民兵、预备役、人民防空建设得到加强。继续做好新形势下的“双拥”工作，加强军民、警民共建，共创全国双拥模范城“五连冠”。外事侨务工作获国侨办表彰，民族、宗教、对台事务等工作继续加强，审计、参事、档案、地方志、保密、老龄和妇女、儿童、残疾人等工作取得了新成绩。

　　各位代表：上述成绩的取得，是省委、省政府正确领导和省直部门大力支持的结果，是市委团结和带领全市上下开拓创新、艰苦奋斗的结果，是方方面面通力协作、全社会共同努力的结果。在此，我代表市人民政府，向为南昌做好“两篇文章”作出重大贡献的全市工人、农民、干部和知识分子，致以崇高的敬意！向各民主党派、工商联、群众团体和社会各界人士，向关心、支持南昌振兴的海内外朋友，向驻昌解放军指战员、武警官兵和广大公安干警，表示衷心感谢！

　　二、当前面临的形势和2003年政府工作任务

　　2003年，是贯彻落实党的十六大精神的重要一年，是全面建设小康社会的开局之年。我国发展正处于一个十分重要的战略机遇期，南昌正面临一个十分有利的宏观环境，机遇与挑战同在，困难和希望并存。东部大发展，中部大崛起，西部大开发，为我们带来了新的机遇；发展不断加快，实力不断增强，环境不断改善，为我们奠定了扎实的基础；社会向心力，事业凝聚力，群众创造力，为我们提供了无穷的力量。

　　但是，我们在前进中还存在着不少困难和问题，主要是：虽然经济增长幅度较高，但总量仍然偏小；虽然优强企业日益增多，但整体实力仍然偏弱；虽然居民收入提高较快，但就业压力仍然较大；虽然发展环境大为优化，但服务意识仍要加强；虽然机关作风有大的改进，但办事效率仍待提高；虽然社会总体稳定，但影响稳定因素仍然不少。对此，我们必须高度重视，采取切实措施，认真加以解决。

　　2003年政府工作的总体要求是：

　　全面贯彻落实党的十六大精神，认真实践“三个代表”重要思想，围绕“富民强市”目标，全力做好“两篇文章”，强力推进“三个率先”，解放思想，实事求是，与时俱进，开拓创新，不断推动经济发展上新台阶、深化改革有新突破、对外开放有新局面、城市建设有新面貌、各项社会事业有新水平、精神文明建设有新成效、人民生活水平有新提高，为全面建设小康社会而努力奋斗。

　　主要预期目标是：

　　国内生产总值620亿元，增长13％；

　　财政总收入69.3亿元，增长14％；

　　地方财政收入30.3亿元，增长106％；

　　全社会固定资产投资178亿元，增长30％；

　　实际利用外资5亿美元，增长38.9％；

　　实际利用内资88亿元，增长35.4％；

　　社会消费品零售总额200亿元，增长11.5％；

　　城市居民年人均可支配收入7500元，增长7.1％；

　　农民年人均纯收入2787元，增长4.5％；

　　人口出生率控制在14.3‰以内。

　　三、以招商引资为重点，力求在全方位对内对外开放上有新突破

　　围绕实现“开放目标更高、开放领域更宽，开放力度更大，开放优势更强”要求，继续坚持“64414”方针，大力突破外资，积极扩大外贸，加速启动外经。特别是紧紧抓住突破外资这个关键，切实做到“三个突出”：一是突出开发区主战场作用，分层次推进各类园区招商引资向深层次发展。以重大项目为突破口，拓展利用外资领域，着力构筑产业高地，形成开放型经济发展的骨架。高新区要着力发展电子、软件、生物医药、精密制造等产业；南昌经济技术开发区要着力发展汽车、化工、机械制造等产业；南昌民营科技园要着力发展食品、包装、服装、轻工等产业。县区开发区要充分利用当地特色资源，建设特色园区，发展特色产业。二是突出开展招商推介活动，提升南昌招商引资的强大吸引力，建立外商代办绿色通道。重大招商活动，精心策划，周密运作；小分队招商，进驻外商密集区，掀起外商投资热，招来好项目，引进大项目，在国内，重点瞄准上海、广东、浙江、福建等发达地区、港澳台地区，承接产业转移，引进各类项目落户南昌。在国外，重点瞄准日本、韩国和东南亚地区，掀起新的招商引资高潮。三是要突出做好项目跟踪服务，尽快形成新的生产力。重点是着力抓好投资大、后劲大的格林柯尔、普天集团、泰丰轮胎、银志服装、香江商贸、金润物流、娃哈哈饮料、TCL数字彩电、奔驰中客、南昌野生动物园等项目的进资、建设、投产。同时，强化重大项目信息报告制度，充分发挥经济“110”快速、高效服务功能，切实帮助外商解决各种困难和问题。

　　四、以工业化为核心，力求在扩充经济总量、提升经济质量上有新突破

　　工业经济主要是着眼构筑南昌新型工业化优势，紧紧围绕“三上两创一提高”（即上规模、上水平、上管理；创新机制、创强后劲；提高南昌工业经济的综合竞争能力），重点抓好“六个一批”：一是建设一批有特色有规模的工业园区，抓好高新产业、经济技术两个开发区的生态工业园建设，提升民营科技园和小兰、昌东、昌南、长?工业园区品牌，加快清华、浙大科技园、先锋国际软件园建设。二是实施一批有后劲的重点技改项目。抓好涂布纸、特种变压器、凯马柴油机、袋式除尘器等技改项目，确保全年完成技改投入16亿元。三是扶持一批市场占有率高的名牌产品。以产品技术质量、市场占有率、规模效益、国际标准认证、出口创汇为重点，提高全顺车、汇仁肾宝、江纺牛仔布系列、金圣卷烟、捷德智能卡等拳头产品的市场占有率，争创中国名牌。四是培育一批在同行业有影响的优势企业。加大对江铃、南烟、南钢、江中等一批重点企业的扶持力度，积极推动一批资产质量高、经济效益好的企业，迅速做大做强，实现倍速增长，有效发挥其带动和推动作用。五是引进一批产业梯度转移项目。全力构建政府服务“高地”，投资成本“洼地”，招商引资“磁场”，吸引MD直升机、中泰凯马等项目早日落户南昌，早日开工建设。六是发展一批专业协作配套企业。建立协作配套信息网络，瞄准大工程、大项目，实施大配套；瞄准大企业、大集团，实施大挂靠；瞄准大市场、大产业，实施大协作。

　　农村经济主要是围绕“建设现代农业，繁荣农村经济，增加农民收入”目标，突出“五个重点”：一是瞄准市场调结构。调优种养品种，调出品种特色，调大特色规模。立足本地资源，发挥比较优势，调整区域结构，形成“一村一品”、“一乡一业”的特色块状经济新格局。引进优良农作物品种，推广经济作物新品种，力争农作物优质品率提高3—4个百分点，经济作物比重达到48％以上。二是培育龙头扩规模。面向市场，建设基地，带动农户。突出抓好农产品批发市场、果品批发市场、河蟹批发市场、水产品市场，不断完善佛塔、温圳、小兰等畜禽批发市场，加快水产品综合交易批发市场、豪威大市场、绿泰农资大市场建设，发挥市场辐射作用，强化龙头企业带动作用，推进农业布局区域化、生产专业化、服务社会化、经营产业化。三是健全体系强服务。突出抓好良种繁育体系建设，大力引进繁育名特优新品种；抓好农技推广体系建设，稳定农技推广队伍，提高农技人员素质；抓好农经信息体系建设，整合信息资源，建立市、县、区、乡信息平台，覆盖全市农村推广使用“一站通”；抓好农产品标准化体系和质量监测体系建设，提高农产品安全生产水平。四是打造品牌增效益。用农业标准化推动农业产业化、品牌优质化，突出开发无公害农产品，打造绿色生态品牌，发展特色传统品牌，提升阳光、孔雀、煌上煌、国鸿等品牌产品的市场占有率。五是转移农民富农民。以推进“小城镇建设工程”为抓手，支持县城和14个省级重点镇及建制镇建设，鼓励乡镇企业向园区集中，吸引农业人口向小城镇集中，使城镇化率达到45％。同时，加强农田水利基本设施建设，组织实施“退耕还林”工程，加大生态环境保护力度，合理开发各类资源。扎实推进农村“双带”致富工程，大力实施“包乡扶村”工程，加快贫困乡村脱贫步伐。

　　第三产业主要是提升商贸流通，发展金融保险，打响旅游品牌，繁荣地产房产。商贸流通重点是以“发展大商贸，建设大市场，搞活大物流”为目标，做好“四创”文章（创新体制、创新科技、创新管理、创新业态），建好三大汽车城（昌北、洪城、红角洲），培育四家大商场（百货大楼、洪城大厦、新洪客隆、北京华联），落实四个大项目（沃尔玛、大润发、百脑汇、欧洲商城）。同时，积极组织会展经济活动，重点做好“第二届南昌金秋经贸活动月”、“第十三届全国厨师节”的组织准备工作。金融保险重点是创新投资融资体制，构建银企交流平台，打造金融营销品牌，创建优良信用环境，扩大企业有效投入，着力实现银企双赢。旅游产业重点是突出“五彩缤纷看南昌”主题，系统性整合各类资源，多渠道筹集建设资金，大手笔开展宣传促销，高标准强化行业管理，争取引进一家国际国内知名的旅游企业集团。房地产业重点是垄断一级市场，繁荣二级市场，土地有序出让，规范房产市场，防止房价过快增长，防止房市出现“泡沫”，确保消费者利益，确保投资者利益。同时，制定优惠政策，建立发展基金，支持优势项目，发展新型三产。

　　五、以产权制度改革为重点，力求在体制机制创新上有新突破

　　一是盘活公有资本。按照“六化”要求（产权关系清晰化、产权主体多元化、产权代表人格化、产权交易市场化、产权保障法制化、劳动用工市场化），实行改制转制、整合重组、转让出售，加快公有资本退出步伐，推进投资主体多元化、经营主体多元化。二是激活民间资本。大力营造发展氛围，重点扶持龙头企业，着力化解发展“瓶颈”，鼓励嫁接国有企业。加大对百户重点个私民营企业扶持力度，建立信贷基金，完善信用担保体系，打造一批“小巨人”，力争30个民营企业年销售收入超亿元。继续发挥民营科技园示范作用，带动昌东工业区、昌南工业园等园区发展。鼓励各类民营经济园区发挥优势，注重特色，使园区产业结构向专、精、特方向发展，形成区域性的企业集群和生产基地。三是用活金融资本。重点是扶持帮助企业上市，力争长力弹簧、洪城水业、晶安高科等3家企业上市融资。四是创新政府职能。重点是调整政府与市场的关系，把资源配置功能交给市场；调整政府与企业的关系，把经营权交给企业；调整政府与事业的关系，启动事业单位机构改革；调整政府与社会的关系，强化政府服务功能。五是健全社保体系。在拓宽就业渠道，增加就业岗位，缓解就业矛盾的同时，强化三条保障线，扩大低保覆盖面，提高低保标准度 ，稳妥推进“下岗保障”向“失业保险”并轨。同时，加快发展残疾人事业，做好残疾人康复、扶贫、培训就业工作。

　　六、以建设现代文明花园英雄城市为目标，力求在推进城市化进程上有新突破

　　继续贯彻“72字”方针（依托一江两岸，展示都市风采，建设花园城市，塑造特色魅力，道路环通成网，建筑各具特色，绿带滨江依湖，园林城中点缀，花卉草坪绿岛，青山绿水夜景，街市繁荣文明，环境整洁优美），画好豫章山水画卷，谱写南昌人文新篇。以创建国家卫生城市和园林城市为目标，认真落实“五高”要求，重点推进“七大工程”：一是城市防洪骨干工程。完成昌南防洪保护圈，组织实施昌北防洪保护线；继续搞好鄱阳湖治理，内外圩堤、大小水库除险加固，涵管枧闸、电排灌站维修改造。二是城市道路骨干工程。构筑中外两环道路骨架，健全城内道路体系，抓好公路联网贯通，完成县乡干道升级改造。实施解放路、昌东大道、高新南大道、洪都中、南大道、红湾公路等新建和改造工程。三是城市环境形象工程。围绕“一江两河八湖”，做足做好山水文章，营造一方新天地，凸现城市新品位。抓好八湖两河工程、八一广场改造、体育公园建设等项目。四是城市公用基础设施工程。污水处理，抓紧实施；大街小巷，管网改造；环卫交通，设施配套；公交车辆，城乡一体；防空防灾，健全体系。启动和完成青山湖污水处理、象湖污水处理、红谷滩污水处理、牛行水厂一期工程、公交停车场、路灯中控系统建设等项目。五是老城区改造工程。重点改造低洼地，拆迁危旧房。做好全市52块低洼地危旧房改造规划，完成30万平方米危旧房改造；全面整治旧城小街小巷，加快“城中村”改造进程。六是红谷滩新区建设工程。加快构筑新城区路网框架，重点突出红角洲建设，迅速提升新区人气。做好前湖文章，做美卧龙山林，高品位推进高校园区建设；新建60万平方米微利商品房，完成90万平方米现代公寓小区住宅；全面启动蝶子湖大型风景旅游区、国际名车展示中心、和泰连锁超市、西奇五星级宾馆、国泰商贸中心等招商引资项目的开工建设。七是长效管理工程。坚持建管并举，理顺管理职能，落实管理责任，完善管理法规，发挥区街作用，推行综合执法。实施“畅通工程”，提高交通管理水平。加强社区建设，拓展社区服务，进一步规范和完善城市社区管理。

　　七、以促进社会全面进步为重点，力求在坚持依法治市、注重以德育人、弘扬社会正气、提高市民素质上有新突破

　　加强民主法制建设。加强行政立法，严格依法行政，强化行政监督，认真实施服务承诺制、政务公开制、首问负责制，简化办事程序，提高工作效率。自觉接受人大法律监督和工作监督、政协民主监督和社会舆论监督，认真办理人大代表议案、建议和政协提案，重视发挥各民主党派、工商联、各群众团体作用，加强政治文明、精神文明、物质文明建设。加强社会治安综合治理，推行社区警务战略，健全防范机制；积极开展严打斗争，严厉打击犯罪行为；强化治安管理，维护社会稳定；褒扬见义勇为，弘扬社会正气。同时，增强国防观念，强化忧患意识；坚持“安全第一、预防为主”的方针，加强安全生产，重视安全消防。优先发展教育。坚持教育创新，增加教育投入，推进管理体制、办学体制、教育体制、人事制度改革；优化教育结构，巩固九年义务教育，普及高中阶段教育，发展地方高等教育。创新科技体制。建立科技创新服务体系，逐步实现服务功能社会化、网络化、产业化。深化科研机构体制改革，多元筹措科研基金，建设科研示范基地，发挥示范带动作用。繁荣文化、报业、广电事业。建立和完善广电集团、报业集团运作机制，推动文化产业发展。加强文化市场管理，繁荣群众文化，丰富市民文化生活。大力实施精品战略，要在“五个一工程”上有突破。加强卫生事业。继续实施卫生“三名”工程，加快城市职工医疗保险体系建设，加强农村卫生和预防保健，深化卫生体制改革，优化卫生资源配置，提升卫生服务能力。广泛开展爱国卫生运动，不断提高人民群众卫生水平。提高体育水平。广泛开展群众性体育运动，大力开发具有地方特色的体育项目，提高竞技体育水平，积极备战全国城运会。稳定低生育水平。夯实基础，强化服务，规范管理，改革创新，控制人口自然增长，提高出生人口素质，促进人口与经济、社会协调发展。

　　八、以转变政府职能、改进机关作风为重点，力求在优质服务、提高效率上有新突破

　　以党的十六大精神为指针，以“三个代表”重要思想为指导，努力建设民主、高效、廉洁、为民的政府。落实机构改革方案，精简公务员队伍，提高公务员队伍整体素质；深化审批制度改革，规范行政审批行为，提高办事效率；积极开展政风评议，加强机关作风建设，克服相互扯皮、推诿拖拉的官僚习气；大力推进阳光工程，促进政务公开，增加政府工作透明度；重视加强信访工作，畅通信访渠道，解决热点难点问题；切实抓好民心工程，突出为民办事，赢得群众信任和支持；完善监督制约机制，加强政府廉政建设，坚持从严治政，坚决惩治腐败；树立求真务实作风，切实搞好服务，推动经济社会快速发展。

　　各位代表，让我们紧密团结在以胡锦涛同志为总书记的党中央周围，务必保持谦虚谨慎、戒骄戒躁的作风，务必保持勤俭节约、艰苦奋斗的作风，乘十六大东风，扬新世纪豪情，解放思想，实事求是，与时俱进，开拓创新，努力实践“三个代表”，全面建设小康社会，伴随着江西在中部地区崛起的步伐，让南昌大展宏图，让南昌再铸辉煌！
